# Adjunctive dexamethasone therapy in unconfirmed bacterial meningitis in resource limited settings: is it a risk worth taking?

**DOI:** 10.1186/s12883-016-0678-0

**Published:** 2016-08-26

**Authors:** Esayas Kebede Gudina, Markos Tesfaye, Aynishet Adane, Kinfe Lemma, Tamiru Shibiru, Andreas Wieser, Hans-Walter Pfister, Matthias Klein

**Affiliations:** 1 Department of Internal Medicine, Jimma University, Jimma, Ethiopia; 2 Centre for International Health, Ludwig-Maximilians-University, Munich, Germany; 3 Department of Psychiatry, Jimma University, Jimma, Ethiopia; 4 Department of Internal Medicine, University of Gondar, Gondar, Ethiopia; 5 Department of Internal Medicine, Hawassa University, Hawassa, Ethiopia; 6 Department of Internal Medicine, Arba Minch Hospital, Arba Minch, Ethiopia; 7 Division of Infectious Diseases and Tropical Medicine, Medical Center of Ludwig-Maximilians-University, Munich, Germany; 8 Department of Bacteriology, Max von Pettenkofer Institute (LMU), Munich, Germany; 9 German Center for Infection Research (DZIF), Partner Site Munich, Munich, Germany; 10 Department of Neurology, Ludwig-Maximilians-University, Munich, Germany

**Keywords:** Bacterial meningitis, Outcome, Dexamethasone, Ethiopia, East-Africa

## Abstract

**Background:**

Bacterial meningitis is associated with significant morbidity and mortality despite advances in medical care. The main objective of this study was to assess the association of adjunctive dexamethasone treatment with discharge outcome of patients treated as bacterial meningitis in low income setting.

**Methods:**

A retrospective study was conducted at four teaching hospitals across Ethiopia. Patients of age 14 years and older treated as cases of bacterial meningitis between January 1, 2011 and April 30, 2015 were included in this study. Information regarding sociodemographic data, clinical presentations, laboratory data, treatments given and status at hospital discharge were retrieved from patients’ medical records using a structured questionnaire. Predefined outcome variables at discharge were analysed using descriptive statistics. Multivariable logistic regression was used to identify factors independently associated with poor outcome.

**Results:**

A total of 425 patients treated with the presumptive clinical diagnosis of bacterial meningitis were included in this study (lumbar puncture done in 56 %; only 19 % had CSF findings compatible with bacterial meningitis, and only 3 % had proven etiology). The overall in hospital mortality rate was 20.2 %. Impaired consciousness, aspiration pneumonia, and cranial nerve palsy at admission were independently associated with increased mortality. Adjuvant dexamethasone, which was used in 50.4 % of patients, was associated with increased in-hospital mortality (AOR = 3.38; 95 % CI 1.87–6.12, *p* < 0.001) and low Glasgow outcome scale (GOS) at discharge (AOR = 4.46 (95 % CI 1.98–10.08). This association between dexamethasone and unfavorable outcome was found to be more pronounced in suspected but unproven cases and in those without CSF alterations compatible with bacterial meningitis.

**Conclusion:**

Most patients treated for suspected bacterial meningitis did not receive proper diagnostic workup. Adjuvant dexamethasone use in clinically suspected but unproven cases of bacterial meningitis was associated with an increased mortality and poor discharge GOS. These findings show that there are potential deleterious effects in unconfirmed cases in this setting. Physicians practising under such circumstances should thus abide with the current recommendation and defer the use of adjuvant corticosteroid in suspected cases of bacterial meningitis.

**Electronic supplementary material:**

The online version of this article (doi:10.1186/s12883-016-0678-0) contains supplementary material, which is available to authorized users.

## Background

Bacterial meningitis is a serious infection of the central nervous system that can progress rapidly and result in death or permanent debilitation [1]. It is associated with a high fatality rate despite advances in medical care [2] and a significant proportion of survivors suffer from long term neurologic sequelae [3]. Most of the cases of acute bacterial meningitis (ABM) occur in low income countries [4] where case fatality rates are higher than in countries with a high standard of medical care [5, 6].

The duration of disease [7], age [8], and immune status of the patient [9–11], timing of antibiotic initiation [12, 13], and type of microorganism [14, 15] were found to be important factors in determining the outcome of ABM. Significant controllable factors known to improve survival and neurologic recovery are rapid diagnosis and an early treatment [12, 16], both of which are difficult to achieve when laboratory support and treatment options are limited [4].

Corticosteroid as adjunctive treatment of ABM is one of the most thoroughly studied and widely discussed controversial issues in recent years [17–21]. Yet, its benefit in mortality and morbidity reduction is far from being settled [20–23]. The existing evidences indicate that the efficacy of dexamethasone varies with etiologic agents [24, 25], clinical circumstances and regions of the world [19, 24–27]. The current adult recommendations limit its use to pneumococcal meningitis in high income countries [24, 25]. Furthermore, the few studies from the developing world did not find any benefits of corticosteroid on mortality and neurologic sequelae [28–30].

We recently analysed the characteristics of 425 patients who were treated and discharged with the presumed diagnosis of bacterial meningitis. One of the main findings was that CSF analysis was done in only 56 % of these patients and the diagnosis could be proven by detection of a causative pathogen in as little as 3.3 % of patients [31]. Now we aimed to assess treatment outcomes and factors associated with poor outcome in these patients. We especially aimed to investigate the effect of adjunctive dexamethasone treatment on the outcome of patients treated for suspected ABM in the four study centres in Ethiopia.

## Methods

### Settings

Ethiopia is a country located in East Africa with an estimated population of 87,952, 000 as of July 2014 [32]. This study was conducted at four teaching hospitals in Ethiopia – Jimma University Specialized Hospital, Hawassa University Referral Teaching Hospital, University of Gondar Hospital and Arba Minch Hospital. The first three are full-fledged university hospitals serving as referral hospitals. Arba Minch hospital is a general hospital affiliated with Arba Minch University’s medical school. All of these hospitals are located in the eastern end of meningitis belt of Africa in different regions of Ethiopia– Northwest (Gondar), southwest (Jimma), south (Arba Minch) and southcentral (Hawassa). The overall catchment population of the four hospitals is nearly 25 million – over a quarter of the Ethiopian population.

### Design

A hospital based retrospective cohort study was conducted using medical record review of patients treated as cases of bacterial meningitis at the four hospitals during the period of 1 January, 2011 to 30 April, 2015. Clinical characteristics of the patients were recently published [31].

### Study participants

Patients included in the study were those of age 14 years and older treated with a presumptive diagnosis of bacterial meningitis and who had complete medical records regarding issues related to diagnosis, treatment and outcome of ABM. Patients whose antibiotic treatment was discontinued before ward admission because of confirmed alternative diagnosis and those with incomplete clinical records were excluded.

Cases treated as bacterial meningitis were categorized based on the 2003 World Health Organization case definition used in WHO-recommended surveillance standards for surveillance of selected vaccine-preventable diseases [33]. These categories are:

- *Suspected unproven cases of bacterial meningitis* – Cases with acute onset (≤7 days) of fever (axillary temperature of ≥38.0 °C) PLUS any of: neck stiffness and altered consciousness PLUS no other alternative diagnosis PLUS no or incomplete CSF analysis.
- *Possible bacterial meningitis* – Cases with clinical signs as described for “suspected unproven ABM” PLUS CSF examination showing at least one of the following three – (1) turbid appearance (2) pleocytosis (>100 white cells/mm^3^) (3) pleocytosis (10–100 white cells/mm^3^) AND either an elevated protein (>100 mg/dl) or decreased CSF to serum glucose ratio (<40 %).
- *Confirmed (proven) bacterial meningitis* – Cases with detected microorganisms by culture, gram stain or agglutination test from CSF specimen.
- *Non-cases* (bacterial meningitis less likely) – Cases not fulfilling any of the above criteria and/or those with evidences suggesting other diagnoses.

### Data collection procedure

Patients treated as cases of ABM were identified using the data from inpatient registration books of medical wards at each hospital. Their medical records were then retrieved from the archives to be reviewed according to a structured questionnaire prepared for the study (Additional file 1). The information gathered included socio-demographic profiles, clinical conditions at presentation, type of antibiotic treatment, adjunctive dexamethasone treatment, clinical course in the hospital, and discharge conditions (death and neurologic sequelae). Glasgow Outcome Scale (GOS) was interpreted from the discharge note (see below).

### Definitions of outcome variables

*Focal neurologic deficit* (FND) – refers to (1) unilateral extremity weakness [monoparesis or hemiparesis] (2) unilateral hypaesthesia (3) localized cranial nerve palsies (III, IV and VII).

*Glasgow Outcome Scale (GOS)* – was the interpretation of treating physician’s documentation of the patient status at discharge. 1 = if death was documented; 2 = if patient was in ‘coma’ or ‘unresponsiveness’ at leaving hospital; 3 = if document included ‘some improvement’ and any of ‘hemiplegia’, ‘paraparesis’, or ‘major disability’; 4 = if document included ‘improved’ with minor sequelae such as ‘facial palsy’ or ‘decreased hearing capacity’; 5 = if document included ‘full recovery’ or ‘discharge with complete improvement’.

*Level of consciousness* – was stated using Glasgow Coma Scale (GCS) which ranges from score of 3 to 15. Patients with score of 15 were considered as fully conscious; 9–14 as *impaired consciousness* and as *coma* for scores between 3 and 8.

### Data processing, analysis and interpretation

The data was checked for completeness and consistency. It was then entered to EpiData version 3.1 and was later transferred to SPSS® (IBM Corporation) version 20 for analysis.

Bivariable analysis was done to identify association between dependent and independent variables. All independent variables with *p* < 0.25 in bivariate analysis were entered for multivariable analysis. Forward logistic regression analysis was done to identify the best fit model. Independent predictors were analysed for three outcome variables – death, Glasgow outcome scale and neurologic sequelae at discharge from the hospital. *P*-values of <0.05 were used as level of statistical significance.

## Results

### Background clinical characteristics

Complete medical records were available for 425 patients treated as bacterial meningitis. The main clinical characteristics of the patients have been previously described in detail [31]. Briefly, the mean age at presentation was 32 ± 15.7 years (range 14 to 85); 52.7 % of them were men. Only about 30 % (127 patients) presented within 2 days of symptom onset. Fever and headache were major presenting symptoms. On presentation, 213 (50.1 %) of patients had impaired consciousness and 33 (7.8 %) had focal neurologic deficits.

HIV infection was detected in 23 (5.4 %) patients, however, only 349 (82.1 %) were tested for it. Forty-four (10.4 %) patients had additional diagnosis of pneumonia, all of which were attributed to aspiration. Lumbar puncture was done for 236 (55.5 %) of patients; 220 (93.2 %) of them had microscopic examination of Gram-stained CSF specimen. Leukocyte count was done for 180 (76.3 %) of these patients but only 58.1 % had analysis for both protein and glucose. Culture was done for only 61(25.8 %) of these cases. Only 14 (3.3 %) of them had a confirmed etiology and classified as “proven ABM”; 82 (19.3 %) belonged to the category of possible ABM. About 46 % (196 patients) were classified as suspected unproven cases. In all cases in this category, CSF was either only partially analysed or not collected at all. The rest 133 (31.3 %) did not fulfil clinical or laboratory based definitions of ABM [31].

Antimeningeal dose of intravenous ceftriaxone (4 g/day) alone or in combination with other antibiotics was used in all patients except for one, who was given a combination of benzyl penicillin and chloramphenicol. Intravenous metronidazole was given to 44 (10.4 %) patients for suspected aspiration pneumonia. Adjunctive dexamethasone treatment was given to 214 (50.4 %) patients [31].

### Discharge outcome

One hundred fifty-six (36.7 %) were discharged with unfavorable outcome (GOS = 1–4); 86 patients (20.2 %) died in the hospital. The median time from hospital admission to death was 3 days; 55.8 % of the deaths occurred in the first 4 days of admission.

Of those who left the hospital alive (339), 277 (81.7 %) were discharged and 57 (16.8 %) left against medical advice (LAMA). Among surviving patients, 70 (20.6 %) had unfavourable GOS (2 to 4); 38 (11.2 %) had documented neurologic sequelae.

The average length of hospital stay (LOS) for discharged patients was 11.0 days (SD = 6.6). Those who left against medical advice had a LOS of 6.1 days (SD = 3.7) and 52.6 % of them left in the first 4 days of admission (Table 1).

**Table 1:** Outcome at leaving hospital in patients treated as bacterial meningitis at teaching hospitals in Ethiopia, 2011–2015

Characteristics	All patients	Adjuvant dexamethasone	No dexamethasone
Status at leaving hospital (*N* = 425), n (%)			
Discharged	277 (65.2)	125 (58.4)	152 (72.0)
Left against medical advice	57 (13.4)	26 (12.1)	31 (14.7)
Referred	5 (1.2)	1 (0.5)	4 (1.9)
Died	86 (20.2)	62 (29.0)	24 (11.4)
Discharge condition of survivors (*N* = 339), n (%)			
Complete recovery	230 (67.9)	98 (64.5)	132 (70.6)
Some improvement	78 (23.0)	41 (27.0)	37 (19.8)
The same as admission	31 (9.1)	13 (8.6)	18 (9.6)
GOS (*N* = 425), n (%)			
1	86 (20.2)	62 (29.0)	24 (11.4)
2	22 (5.2)	9 (4.2)	13 (6.2)
3	9 (2.1)	5 (2.3)	4 (1.9)
4	39 (9.2)	26 (12.1)	13 (6.2)
5	269 (63.3)	112 (52.3)	157 (74.4)
Neurologic sequelae (*N* = 38 ^a^), n (%)			
Low GCS	18 (47.4)	5 (27.8)	13 (59.1)
Hemiparesis	8 (21.1)	4 (22.2)	4 (18.2)
Seizure	9 (23.7)	6 (33.3)	3 (13.6)
Paraparesis	3 (7.9)	3 (16.7)	0
Cranial nerve palsy	2 (5.3)	0	2 (9.1)
Length of hospital stay in days, mean (SD)			
Total	8.9 (6.4)	8.8 (7.0)	9 (5.8)
Discharged patients	11.0 (6.6)		
LAMA	6.1 (3.7)		
Referred	7.4 (5.2)		
Died	4.0 (3.4)		

*GOS* Glasgow outcome scale, *LAMA* left against medical advice

^a^2 Patients had multiple complications

### Factors associated with poor outcome

*Glasgow outcome scale (GOS)* – by dichotomizing GOS into favorable (GOS = 5) and unfavorable (GOS =1 to 4) outcome, we found that admission GCS (AOR = 0.77; 95 % CI = 0.66–0.89) and dexamethasone treatment (AOR = 4.46; 95 % CI 1.98–10.08) were independently associated with unfavorable outcome. Note that GCS had reverse association with poor outcome; every increment from lowest of 3 to 15 resulted in improvement of outcome by 23 %.

Fifty-two (12.2 %) of patients were additionally treated with presumptive diagnosis of tuberculous meningitis (TBM). These groups of patients had unfavourable outcome at discharge as compared to other groups (AOR = 2.78; 95 % CI 1.06–7.30).

*In hospital death* – Admission Glasgow coma scale, presence of pneumonia and cranial nerve palsy during hospitalization were patient related factors independently associated with increased mortality. Accordingly, every drop of GCS from 15 was associated with increment of mortality by 21 % (AOR = 0.79; 95 % CI = 0.73–0.85). On the other hand, adjunctive dexamethasone therapy was found to be associated with over 3 times increment of mortality (AOR = 3.38; 95 % CI = 1.87–6.12) (Table 2). However, no association was seen between increased mortality and other conventional risk factors such as duration of illness, age of the patient and HIV infection.

**Table 2:** Factors independently associated with poor outcomes at leaving hospital in patients treated as bacterial meningitis at teaching hospitals in Ethiopia, 2011–2015

Variable	AOR	95 % CI	*P*-value
Death			
Level of consciousness at presentation (*for a point increase in GCS*)	0.79	0.73–0.85	<0.001
Dexamethasone treatment	3.38	1.87–6.12	<0.001
Aspiration pneumonia at presentation	2.97	1.36–6.41	0.006
Cranial nerve palsy at presentation	4.73	1.45–15.50	0.010
Unfavorable outcome (GOS = 1–4)			
Level of consciousness at presentation (*for a point increase in GCS*)	0.77	0.66–0.89	<0.001
Dexamethasone treatment	4.46	1.98–10.08	<0.001
TB suspected cases	2.78	1.06–7.30	0.038
Neurologic sequelae			
Focal neurologic deficit at presentation	3.33	1.31–8.50	0.012
Seizure at presentation	2.20	1.03–4.67	0.041
Duration of illness before presentation	1.09	1.01–1.16	0.020
Impaired consciousness (GCS < 15)	2.65	1.21–5.81	0.015

This table presents output of Forward logistic regression analysis

*Neurologic sequelae* – Focal neurologic deficits (AOR = 3.33; 95 % CI 1.31–8.50), seizures (AOR = 2.20; 95 % CI 1.03–4.67) and a low level of consciousness (AOR = 2.65; 95 % 1.21–5.81) at admission were associated with the occurrence of neurologic sequelae at discharge. This analysis showed also that a delay of one day from symptom onset to hospital presentation was associated with 9 % increment in risk of neurologic sequelae (AOR = 1.09; 95 % CI 1.01–1.16) (Table 2).

As described above, 15 % of patients left hospital against medical advice or referred for better care. Separate analysis was done to assess if these patients differed clinically from discharged patients. Accordingly, they were found to have lower GOS, lower GCS and higher proportion of neurologic sequelae when leaving the hospital (Table 3).

**Table 3:** Difference in secondary outcome variables between discharged patients and those who left the hospital against medical advice or were referred to other centres, in patients treated as bacterial meningitis at teaching hospitals in Ethiopia, 2011–2015

Discharge outcome	GOS, N (%)					Neurologic sequelae, N (%)			Impaired GCS, N (%)		
	2	3	4	5	*P*	Yes	No	*P*	Yes	No	*P*
LAMA/referred	21 (33.9)	6 (9.7)	20 (32.3)	15 (24.2)	<0.001	16 (25.8)	46 (74.2)	<0.001	13 (21.0)	49 (79.0)	<0.001
Discharged	1 (0.4)	3 (1.1)	19 (6.9)	254 (91.7)		18 (6.5)	259 (93.5)		2 (0.7)	275 (99.3)	

### Dexamethasone treatment and its association with discharge outcomes

Chi-square test showed that dexamethasone was used more often in confirmed and probable cases of bacterial meningitis, those with turbid CSF and organism detected by Gram staining. On the other hand, it was found to be prescribed less often in HIV-positive patients as compared to non-HIV cases. There was also a clear difference in the pattern of dexamethasone treatment between hospitals ranging from 23.5 % at Hawassa to 73.9 % at Gondar (Table 4).

**Table 4:** Comparison of background characteristics by dexamethasone treatment of patients treated as bacterial meningitis in Ethiopia, 2011–2015

Characteristics	Dexamethasone	No dexamethasone	*P* value
Mean age, year (SD)	30.1 (15.0)	33.9 (16.2)	0.116
Duration of illness, days (SD)	5.2 (4.3)	5.0 (4.3)	0.683
Diagnosis of meningitis			
Confirmed	12 (85.7)	2 (14.3)	0.001*
Probable	51 (62.2)	31 (37.8)	
Suspected	84 (42.9)	112 (57.1)	
Non-cases	67 (50.4)	66 (49.6)	
Prior antibiotic treatment			
Yes	45 (43.3)	59 (56.7)	0.096
No	169 (52.6)	152 (47.4)	
Impairment of consciousness			
Yes	102 (52.0)	94 (48)	0.52
No	112 (48.9)	117 (51.1)	
Focal neurologic deficit			
Yes	22 (66.7)	11 (33.3)	0.051
No	192 (49.0)	200 (51.0)	
CSF appearance			
Turbid	37 (64.9)	20 (35.1)	<0.001*
Normal	68 (38.2)	110 (61.8)	
Detection of organism by Gram stain			
Yes	11 (84.6)	2 (15.4)	0.003*
No	89 ((43.0)	118 (57.0)	
HIV status			
Positive	7 (30.4)	16 (69.6)	0.048*
Negative	207 (51.6)	194 (48.4)	
Hospital			
Jimma	52 (42.6)	70 (57.4)	<0.001
Gondar	24 (26.1)	68 (73.9)	
Hawassa	65 (76.5)	20 (23.5)	
Arba Minch	73 (57.9)	53 (42.1)	

*statistically significant

**Fig. 1:**
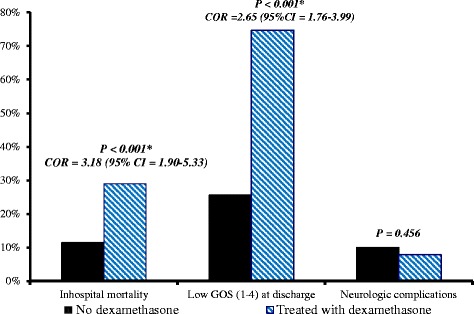
Association between adjuvant dexamethasone treatment and discharge outcomes in patients treated as bacterial meningitis in Ethiopia, 2011–2015. COR – Crude odds ratio, GOS – Glasgow Outcome Score. * Denotes statistical significance

Dexamethasone treatment was associated with an increase of the in-hospital mortality, COR = 3.18 (95 % CI 1.90–5.33); *p* < 0.001 and low GOS at discharge, COR = 2.65 (95 % CI 1.76–3.99); *P* < 0.001. However, there was no association with neurologic sequelae at discharge (Fig. 1).

As depicted on Table 2, dexamethasone was found to be one of the factors independently associated with poor outcome on multivariable analysis with the best-fit model. When further analysis was performed controlling for all potential confounders on multiple logistic regressions with forced entry, this association persisted. For instance, the odds of having low GOS at discharge was nearly 4 times, AOR = 3.94 (95 % CI 1.63–9.53; *P* = 0.002) and its association with in hospital death was also nearly as much, AOR = 3.60 (1.97–6.60; *P* < 0.001).

Controlling for these individual variables also revealed similar finding as shown on Table 5.

**Table 5:** Subgroup analysis for association of dexamethasone with discharge outcome in patients treated as bacterial meningitis at teaching hospitals in Ethiopia, 2011–2015

Variables	Death			Low GOS		
	OR	95 % CI	*P*	OR	95 % CI	*P*
Age						
< 50 years	3.08	1.74–5.48	<0.001*	2.83	1.79–4.48	<0.001*
> = 50 years	4.00	1.19–13.46	0.025*	2.68	1.0–7.21	0.051
Duration of illness						
< =2 days	4.02	1.37–11.77	0.011*	2.05	0.94–4.50	0.072
> 2 days	2.93	1.62–5.30	<0.000*	2.91	1.79–4.72	<0.001*
Level of conscious						
14 and 15	2.21	0.94–5.21	0.070	1.82	0.996	0.051
< 14	3.69	1.84–7.40	<0.001*	388	20.3–7.44	<0.001*
HIV status						
Positive	3.25	0.46–22.93	0.237	18.00	1.63–198.51	0.018*
Negative	3.38	1.91–5.66	<0.001*	2.49	1.63–3.80	<0.001*
Prior antibiotics						
Yes	3.59	1.24–10.38	0.018*	5.97	2.47–14.44	<0.001*
No	3.04	1.68–5.50	<0.001*	2.07	1.30–3.29	0.002*
Focal neurologic deficit						
Yes	3.20	0.67–15.38	0.146	0.80	0.16–3.99	0.789
No	3.00	1.72–5.23	<0.001*	2.77	1.80–4.29	0.003*
CSF appearance						
Turbid	2.72	0.67–11.11	0.163	2.44	0.79–7.51	0.121
Clear	6.26	1.95–20.12	0.002*	4.28	2.14–8.58	<0.001*
TB suspected						
Yes	3.90	0.45–34.02	0.218	3.46	0.83–14.36	0.087
No	3.09	1.79–5.34	<0.001*	2.18	1.40–3.39	0.001*
Diagnosis of meningitis						
Confirmed	–	1	1	–	1	1
Probable	2.31	0.68–7.86	0.180	3.22	1.24–8.36	0.016*
Suspected	3.38	1.73–6.59	<0.001*	2.09	1.16–3.75	0.014*
Non-cases	6.98	1.50–32.56	0.013*	3.17	1.41–7.15	0.005*

*Statistically significant

*Dexamethasone and mortality* – This association did not differ between older and younger patients, whether a patient had delayed presentation or not, and whether a patient took prior antimicrobial treatment or not. However, this association faded in HIV infected patients, those with neurologic deficit on presentation, those with abnormal CSF findings and TB suspected cases.

*Dexamethasone and low GOS* – Age of the patient, presence or absence of HIV infection, and prior antibiotic treatment did not change the nature of this association. On the other hand, the association disappeared in TB suspected cases, those with confirmed ABM and neurologic deficit on presentation.

In summary, adjunctive dexamethasone treatment was associated with poor outcome in most of the sub-groups. However, this was not the case in patients with a diagnosis of bacterial meningitis that was microbiologically proven or supported by CSF findings (those with turbid CSF, proven BM and probable cases of BM), and in those suspected to have TBM. In these cases, dexamethasone therapy was not associated with any positive or negative discharge outcomes (Table 5).

The only one instance where dexamethasone was associated with positive outcome is decreased discharge neurologic sequelae in patients who had impaired consciousness at presentation, AOR = 0.42 (95 % CI 0.19–0.94; *P* = 0.033).

## Discussion

Our study revealed that patients treated for clinically suspected ABM in teaching hospitals in Ethiopia were found to have a high mortality of 20 %. However, most of these patients did not receive a proper diagnostic workup and were treated only pragmatically [31]. Hence, the reported mortality may not reflect the true burden of the problem in the settings. Moreover, adjunctive dexamethasone treatment in patients who did not receive proper CSF analysis or in whom CSF findings were not compatible with acute bacterial meningitis was associated with poor discharge outcomes.

The finding in our study may not reflect the real mortality associated with ABM in the setting. This is because the diagnosis of ABM in substantial fraction of the patients was unclear and it is likely that many of these patients suffered from diagnosis other than ABM [31]. Therefore, it is not possible to compare the data to other studies in patients with proven acute bacterial meningitis. It further explains why the mortality of 20 % is much lower than in meningitis studies from other low income countries like Malawi, where mortality reached 40 % [5, 6]. This is also reflected in the documented short-term neurologic sequela in survivors which was only 11.2 %, a rate that is lower than in most reports on the outcome of ABM [3, 5, 6, 15, 34, 35].

As expected, impaired consciousness at admission was associated with poor outcome. This may be due to the severity of the illness as well as the occurrence of complications like aspiration pneumonia which on itself was associated with a higher mortality. However, conventional poor prognostic indicators like age, causative bacteria, duration of illness, and HIV infection were not found to be associated with adverse outcome. The study was not able to detect an effect of these factors due to small number of confirmed cases and due to the fact that only the outcome at leaving the hospital was assessed. The study may also be underpowered to detect an association because of the small number of HIV cases. However, 17.9 % of patients did not receive HIV test which might have underestimated its real prevalence.

Adjuvant corticosteroid in management of bacterial meningitis in low and middle income countries, and in settings with high HIV prevalence in particular, has never been proven beneficial [28–30]. To date, there is no recommendation of its use in such settings. However, physicians in settings with little evidence and diagnostic facilities like Ethiopia have continued its use based on recommendation for high income countries [36, 37]. The finding in our study, where dexamethasone was used in half of the patients, is a testimony of scepticism towards the current recommendation in low income countries.

As it highlighted on Table 5, dexamethasone use was not associated with any positive or negative outcome in those patients with confirmed or probable cases of bacterial meningitis. The lack of evidence for its benefit in Ethiopia is consistent with previous findings from similar settings [28–30], although the number of patients with proven acute bacterial meningitis was too low to address this question.

More important, however, this study demonstrated that dexamethasone treatment seems to be harmful in patients who were treated as ABM without any CSF findings that supported the diagnosis of ABM or when CSF was not analysed. One likely explanation for its association with unfavorable outcome is that, as it has repeatedly been highlighted in this paper, the majority of the patients likely suffered from other alternative diagnoses. Dexamethasone administration to patients with severe brain or systemic infections without simultaneously treating the underlying disease condition could have resulted in the poorer outcomes. As this is the first study that looked at the effect of dexamethasone in patients treated with only clinically presumptive diagnosis of ABM, it makes it valuable for the real situation in developing countries.

However, our study has multiple limitations worth mentioning. This is a retrospective study which may be affected by poor documentation and loss of medical records. Moreover, patients who were given dexamethasone might have had severe disease from the outset and, hence, the poor outcome could be due to their underlying medical condition rather than the dexamethasone use per se. Last but not least, the study may not be representative for the country because it involved mainly teaching hospitals. Nevertheless, it must be assumed that the rate of treatment of cases with suspected ABM lacking any diagnostic workup is even higher throughout the country.

## Conclusion

Adjuvant dexamethasone use in management of suspected but unproven cases of bacterial meningitis in teaching hospitals in Ethiopia was associated with an increased mortality and poor discharge GOS. These findings re-affirm the lack of evidences for its broad use for presumed meningitis in low income countries and show that there are potential deleterious effects in unconfirmed cases. Physicians practising under such circumstances should abide with the current recommendations and defer the use of adjuvant corticosteroid in clinically suspected cases of bacterial meningitis without CSF alterations that support the diagnosis.

## Additional file

Additional file 1: Assessment of treatment strategies for bacterial meningitis in Ethiopia. The English version of the tool used for data collection. (PDF 836 kb)

## Abbreviations

ABM: Acute bacterial meningitis
AOR: Adjusted odds ratio
CI: Confidence interval
COR: Crude odds ratio
CSF: Cerebrospinal fluid
GCS: Glasgow coma scale
GOS: Glasgow outcome scale
HIV: Human immunodeficiency virus
LAMA: Left against medical advice
LOS: Length of (hospital) stay
OR: Odds ratio
SD: Standard deviation
TBM: Tuberculous meningitis
WHO: World health organization
