# Supplementary material for: Adjunctive dexamethasone therapy in unconfirmed bacterial meningitis in resource limited settings: is it a risk worth taking?
Source: BMC Neurol. 2016 Aug 26;16(1):153. doi: 10.1186/s12883-016-0678-0 (PMC5000408; doi:10.1186/s12883-016-0678-0)
Supplement: Additional file 1: — Assessment of treatment strategies for bacterial meningitis in Ethiopia. The English version of the tool used for data collection. (PDF 836 kb) [file 12883_2016_678_MOESM1_ESM.pdf]

I. Identification

1. Initials _____________
2. MRN_______________
3. Hospital____________
4. Age_________________________
5. Gender______________________
6. Date admitted_______________
7. Area of residence
   a. Urban ☐
   b. Rural ☐

II. Clinical history at presentation

8. Duration of illness (days)_____________ or hours____________
9. Symptoms (check all that apply)
   a. Fever☐
   b. Headache☐
   c. Neck rigidity☐
   d. Vomiting ☐
   e. Photophobia ☐
   f. Loss of consciousness ☐
   g. Seizure ☐
   h. Body weakness☐
   i. Skin rash ☐
10. Any documented risk factor or underlying disease (check all that apply)
    c. Smoker☐
    d. Alcoholic ☐
    e. Diabetes mellitus☐
    f. Pregnancy☐
    g. Known HIV patient☐
    h. Similar illness at home or in the vicinity☐
    i. Other, specify ___________________________
11. Antibiotic treatment for the same complaint before presentation:
    a. Yes☐
    b. No ☐
12. If yes, mention the **type** and **route** of administration

    ____

    ____
13. If yes, **when** and for **how long** was the treatment given? (days)___________________
14. If yes, **where** was the treatment given
    a. Health centre ☐
    b. Private clinic ☐
    c. Other hospital ☐
    d. At this hospital ☐
    e. Other, specify_____________
    f. Not specified ☐

III. Physical examination findings at presentation

15. Vital signs
    a. T (^{0}C)________
    b. PR (bpm)_________
    c. RR (per minute) _________
    d. BP (mmHg)_________
16. GCS_____________

17. Cranial nerve palsy (III, VI, VII)
    a. Yes, specify____
    b. None ☐
18. Hemiparesis/hemiplegia
    a. Yes☐ b. No☐

IV. Laboratory findings

19. Was LP done
    a. Yes ☐ b. No ☐
20. If **no**, mention the reason____

If yes to Q19, ANSWER Q21 THROUGH 30. If NO, go directly to Q31

21. CSF Appearance
    a. Crystal clear☐
    b. Turbid☐
    c. Bloody ☐
22. **A**. CSF Glucose (mg/dl)__________ **B**. CSF glucose to serum glucose ratio________
23. CSF Protein (mg/dl)___________
24. CSF cell count (per HPF)___________PMN(%)________Lymphocyte (%)________
25. Gram stain finding
    a. Positive☐ b. Negative☐ c. Not done☐
26. If positive, give all the descriptions here ________________________________

    ____

    ____
27. Indian ink
    a. Positive☐ b. Negative☐ c. Not done☐
28. ZN stain for AFB
    a. Positive☐ b. Negative☐ c. Not done☐
29. CSF culture and sensitivity test?
    a. Positive ☐ b. Negative☐ c. Not done ☐
30. If positive, give the type of organism and their sensitivity pattern

    ____

    ____

Complete Blood count (CBC) – Q31-33

31. WBC (per µl)______

    Neutrophils (%) __ Lymphocytes (%) __ Others cells (%) __

32. Hb(g/dl)_____

33. Platelet (per µl)______

34. ESR __________________mm/in the first hour

35. Blood glucose (mg/dl)________

36. HIV rapid test

    a. Positive☐ b. Negative ☐ c. Not done☐

Answer Q37 to 40 if HIV positive, if not, go directly to Q41

37. What is the current WHO stage? _______

38. What is the CD4 count? ________________

39. Is the patient on HAART?

    1. Yes ☐ 2. No ☐

40. If on HAART, for how long? (years) ________________OR months_____________

41. Blood film for haemoparasite

    a. Positive☐ b. Negative☐ c. Not done☐

    Mention the finding if positive____________________________________

V. Treatment

42. Mention the **regimen**, **route**, **dose** and **duration** of antibiotic treatment

    ____

    ____

    ____

43. Was dexamethasone given

    a. Yes☐ b. No ☐

44. If there is additional treatment given for additional or underlying disease

    ____

    ____

    ____

VI. Outcome assessment

45. What was the outcome at leaving service?

    a. Improved☐ b. Died☐ c. Left against medical advice☐

46. Total stay in hospital in **days** ___________________or **hours**__________________

Data accuracy and completeness is essential for evidence based practice!

47. GOS at leaving hospital__________________________(*please complete the details on separate questionnaire for GOS)*
48. Symptoms at discharge if any____

    ____
49. Discharge neurologic sequalae if any______________________________________

    ____
50. If there are other meningitis related complications document at discharge, give it here____

    ____
51. Complete discharge assessment
    a. Complete improvement☐
    b. Some improvement☐
    c. No improvement☐
52. Is discharge diagnosis different from admission?
    a. Yes☐ b. No☐
53. If yes to Q52, give full diagnosis at discharge_______________________________

    ____
54. In case of in hospital death, what was the possible immediate cause of **death**?
    a. Brain herniation☐
    b. Respiratory failure☐
    c. Septic shock ☐
    d. Multisystem organ failure☐
    e. Other, specify____

       ____

Completed by________________________ Sign.______________ Date____________
Checked by _________________________ Sign. ______________Date ____________

GLASGOW OUTCOME SCALE

Initials: ____________________________ MRN_________________

Age: ____________________________ Gender_________________

Score Description

1. DEATH
2. PERSISTENT VEGETATIVE STATE

   Patient exhibits no obvious cortical function.
3. SEVERE DISABILITY (Conscious but disabled).

   Patient depends upon others for daily support due to mental or physical disability or both.
4. MODERATE DISABILITY (Disabled but independent).

   Patient is independent as far as daily life is concerned. The disabilities found include varying degrees of dysphasia, hemiparesis, or ataxia, as well as intellectual and memory deficits and personality changes.
5. GOOD RECOVERY

   Resumption of normal activities even though there may be minor neurological or psychological deficits

TOTAL (1–5): ______

Completed by________________________ signature _________________date_________

Checked by_________________________signature__________________date_________
